# Role Centrality and Shared Activities With Grandchildren: Effects on Grandparent Depression

**DOI:** 10.1093/geroni/igaa057.1121

**Published:** 2020-12-16

**Authors:** Madeline Marello, Julie Patrick

**Affiliations:** West Virginia University, Morgantown, West Virginia, United States

## Abstract

Research shows that physical and mental health are closely linked (Ohrnberger, Fichera, & Sutton, 2017). Further, social role theory states that holding and enacting valued roles, such as grandparenting, can buffer the negative effects of health on depression (Reitzes & Mutran, 2004). Using data from 247 grandparents (Mean age = 66.5; range 42 to 90 years; 46.2% grandfathers), we examined the differences between 164 custodial and 83 traditional grandparents on whether grandparent role centrality and engagement with grandchildren altered the effects of physical health on depression. The multigroup moderated moderation model was significant (X2(DF=30, N = 247) = 1610.78, p < .001; R2 = .797). We examined whether the paths were moderated by custodial status. Among custodial grandparents, role centrality (β = -.482**) and shared activities (β = -.493***) were significant predictors of depressive symptomatology. Moreover, the interaction between physical health and activities (β = .488***) and between physical health and role centrality (β = .522**) also accounted for significant variance among custodial grandparents. Custodial grandparents in poorer health who valued the grandparent role and those in poorer health who engaged with their grandchildren experienced fewer depressive symptoms. No such patterns were observed for traditional grandparents. Although we had anticipated that the interaction between role centrality and engagement with grandchildren would predict depressive symptoms, the interaction did not reach significance. Results are discussed in terms of the need to examine the differences of family/social contexts in grandparent populations.

